# Educational Intervention Boosts Dietitians’ Knowledge of the Safety and Approval of Low- and No-Calorie Sweeteners

**DOI:** 10.3390/nu17010032

**Published:** 2024-12-26

**Authors:** Alissa A. Nolden, James Makame

**Affiliations:** Department of Food Science, University of Massachusetts Amherst, Amherst, MA 01003, USA

**Keywords:** registered dietitian, nutritionist, artificial sweeteners, safety, dietary guidance, education

## Abstract

Background/Objectives: Low- and no-calorie sweeteners (LNCSs) provide sweetness in food applications that are suggested to support consumers’ reduced consumption of caloric sweeteners and added sugar intake. Consumers seek guidance and advice on using LNCSs from healthcare providers, including dietitians and nutritionists. However, prior research suggests there may be inconsistent guidance on the use of LNCSs. The main goal is to assess dietitians’ and nutritionists’ perceptions and knowledge of LNCSs. We also evaluate the impact of educational intervention on the perceptions and knowledge of low- and no-calorie sweeteners (LNCSs) among healthcare professionals. Methods: Participants completed an online questionnaire on their perceptions of LNCSs before and after viewing a webinar given by a member of the FDA on the safety and approval process of LNCSs. A total of 187 participants completed the pre-questionnaire, and 58 participants completed the post-questionnaire. Results: The LNCSs most familiar to participants was aspartame, followed by stevia, with thaumatin, advantame, and neotame being the least familiar sweeteners. While all sweeteners were FDA-approved, there were differences in perceived safety and suitability for dietary use. Following the intervention, there was a significant improvement in the participant’s knowledge, understanding, and confidence in the safety and approval process of LNCSs and reduced negative perceptions of LNCSs on health. Conclusions: With nutritionists and dietitians being the primary sources of nutritional guidance, consumers may seek their recommendations regarding using LNCSs. However, this study revealed differences in suitability across FDA-approved LNCSs and a lack of confidence regarding the safety and approval process. Education programs regarding the safety and approval process of LNCSs increase confidence in advising patients and clients on the use of LNCSs.

## 1. Introduction

The World Health Organization (WHO) recommends reducing free sugar intake to less than 10% of daily energy intake [1,2,3]. However, in many parts of the world, sugar intake remains high. Canadians consume about 12% (range: 11–16%) of their energy from free sugar [4], and among Africans, especially in the middle- to high-income countries, free sugar energy intake rates are estimated to be between 10.5% and 14.8% [5]. Similarly, in the U.K. and U.S., free sugar energy intake values remain much higher than recommendations, at 12.4% [6] and 16.3% [7], respectively. These high levels of sugar intake are an international health concern, and the resulting high intake is associated with increased economic burden.

Low- and no-calorie sweeteners (LNCSs), defined as molecules with a sweetness profile at least 30 times higher than sucrose [8], have been positioned as a potentially viable strategy for reducing dietary sugar intake [9,10,11]. However, there is a discrepancy between the literature and the guidelines for their use [12,13]. This inconsistency in recommendations may drive uncertainty about using LNCSs among healthcare providers. Nonetheless, the food and beverage industry has focused on using LNCSs to deliver sweetness in products to support the reduction or removal of caloric sweeteners. LNCSs have been referred to as high-intensity or high-potency sweeteners. However, it is not that they are more intense than sucrose; it takes a minimal amount of LNCS to produce a sweet sensation compared to caloric sweeteners, like sucrose [14,15]. Broadly classified as either natural or artificial, LNCSs differ in chemistry (e.g., some are aldehydes, ketones, sugar alcohols, proteins, amino acids, or peptides). LNCS are suggested to have added consumer benefits, including reduced free sugar intake, reduced calories, and a lower glycemic impact [16] with no long-term metabolic effects [17] (see review [15]). Their inability to stimulate insulin secretion makes LNCSs an option for diabetic patients, allowing them to enjoy the sweetness of foods and beverages [15,18,19]. In a meta-analysis of 20 studies, Laviada-Molina and colleagues (2020) concluded that using LNCSs was associated with a significant reduction in body weight and BMI among overweight and obese consumers [20]. Indeed, there is a growing focus on LNCSs and their use in supporting nutrition-related public health objectives linked to reducing dietary sugar intake, including weight control, obesity, dental caries, cardiovascular diseases, and type 2 diabetes [21,22,23].

However, most consumers do not fully understand LNCS and may be unsure or apprehensive about using them to replace caloric sweeteners [24]. Considering that healthcare professionals (HCPs) are an essential resource for adults seeking dietary guidance, it is important to understand HCPs’ perceptions of LNCSs. HCPs include dietitians, nutritionists, nurses, physicians, nurse aids/physician assistants, medical doctors, and others, often working in public or private healthcare institutions. Indeed, adults in the US report trusting their HCPs to provide information regarding LNCS and distrust bloggers and social media influencers, whom they consider to be nonexperts [25]. Even though HCPs are viewed as experts regarding LNCSs, it is not well understood how HCPs view the safety and use of LNCS. In the present study, recruitment efforts were made to enroll a diverse population of HCPs; however, the final population resulted in a majority of registered dietitians, limiting the ability to generalize to the targeted audience of HCPs. Nonetheless, dietitians and nutritionists are a primary source of nutrition-related guidance, especially among diabetic and obese individuals [26,27].

There has been limited investigation in assessing HCPs’ perceptions of LNCSs, with three studies surveying HCPs [28,29,30]. In our review of these studies, a total of 312 HCPs combined across studies were evaluated from 6 different countries. Table 1 reports a summary of the findings from these studies identified. Overall, the findings suggest that HCPs describe concern about the available evidence of the scientific data regarding the health implications and safety of LNCSs. As a result, this perceived lack of evidence has led HCPs to consider LNCS as a high-risk for use in diets [30]. Sollid, Webster [31] proposed that this lack of trust or understanding of the regulatory processes and approval process could fuel the negative perceptions of LNCSs. Farhat and colleagues came to a similar conclusion, noting that a lack of knowledge of LNCSs is due to not having access to objective data on the risk–benefit profiles of LNCSs nor being familiar with how they are regulated [30]. While these studies provide important insights, HCPs from the U.S. were not represented.

Continuing educational programs have been an effective strategy for increasing knowledge and education among HCPs [32,33,34]. Regarding the education of nutrition and dietary guidance, it has become apparent that HCPs, including dietitians and nutritionists, do not receive adequate education [35,36]. Considering this, and the fact that there is low awareness regarding the safety and regulatory processes of LNCSs, we propose that education on the safety and regulatory review process may influence perceptions regarding LNCS. Therefore, the present study investigates whether educational material (i.e., intervention) on the regulatory approval process influences the perceptions of LNCSs among dietitians and nutritionists in the U.S.

The overall objective of the present study is to (i) assess perceptions of LNCSs and (ii) assess the effectiveness of an educational intervention in increasing confidence regarding recommendations and the safety of LNCSs. This information will help to establish a better understanding of the confidence and knowledge regarding LNCS and provide important insights into the effectiveness of educational programming. Increased education and confidence regarding LNCS use can better equip providers to support patients who require dietary counseling.

## 2. Materials and Methods

### 2.1. Overview

Individuals identified as healthcare providers were invited to participate in this research study. After obtaining consent, participants completed a pre-questionnaire, followed by an educational intervention where participants viewed a 1 h webinar and completed a post-questionnaire. All study activities were completed online and on participants’ personal devices. The survey and the video were hosted on Caspio (Sunnyvale, CA). After completing the research study, participants could receive continuing education credits (CEUs). The CEUs are approved for the Commission on Dietetic Registration provided by the Institute for the Advancement of Food and Nutrition Science (IAFNS). The Institutional Review Board approved this study at the University of Massachusetts Amherst (protocol#: 4597).

### 2.2. Recruitment

Interested individuals were eligible to participate if they met the following criteria: at least 18 years of age, practicing or practiced in the United States, and willing to complete two online questionnaires and view a 1 h webinar on the approval process of LNCSs. This research study recruited individuals through flyers, social media, and HCP channels such as membership organizations and associations for family practitioners, nurses, dietitians, and posting to websites (e.g., IAFNS).

A total of 187 individuals completed the pre-questionnaire, with 58 participants completing both the pre- and post-questionnaires. Prior to analysis, one individual was removed for being of an invalid age. From the pre-questionnaire, there were 187 participants with a mean age of 43.5 (±13.8), with the majority (over 91%) being registered dietitians (RDs). Others reported being nutritionists (n = 5), registered dietetic technicians (n = 4), MDs (n = 2), nutrition scientists (n = 3), and other (n = 1). The participants reported their primary patient population, with the majority of participants working with adult (n = 55), geriatric (n = 15), adult and geriatric (n = 36), or all age groups (n = 46), with fewer reporting pediatric (n = 17) or pediatric and adult (n = 7) patients. Some participants reported that they were not currently seeing patients (n = 13). The majority of the study population reported having more than 20 years experience (n = 59), followed by 11–20 years (n = 47), 5–10 years (n = 38), and 1–5 years (n = 37), with few participants having less than 1 year experience (n = 6).

### 2.3. Study Design

#### 2.3.1. Questionnaire

This study consisted of two online questionnaires: one completed before (pre-) and one completed after (post-) viewing the video. Researchers and healthcare providers reviewed these questionnaires. Based on prior studies [28,29,30] and knowledge assessment tools [37], questions were selected and revised as needed. Participants were first asked to report their age, choose their primary area of practice, and indicate the age group of their patient group.

Next, participants were asked to select their sources of information or training related to new nutritional science and research. Possible selections included professional association meetings and conferences, formal educational courses through a university, continuing education courses/seminars/webinars, trade association communications, key opinion leaders in your network of healthcare professionals, peer-reviewed journals, and social and other traditional media reports. For each source selected, participants were asked to rate the perceived trustworthiness on a 5-point Likert scale with end anchors on the two poles: not at all trustworthy (1) and very trustworthy (5).

The remainder of the questions were specific to their experiences, perceptions, and self-reported knowledge of LNCS. Participants were asked to report their understanding of LNCSs, their confidence regarding their safety and approval process, and their agreement with several questions/statements relating to LNCS use. There were three questions where participants were asked to select the LNCS that they (i) were currently familiar with; (ii) believed to be safe and had sufficient safety data; and (iii) believed to be unsuitable or lacking safety data. LNCS in this study included acesulfame potassium (Ace-K), advantame, allulose, aspartame, monk fruit, neotame, saccharin, stevia, sucralose, thaumatin, and sugar alcohols or polyols (e.g., xylitol, erythritol). Participants had the option of selecting all of these, any FDA-approved sweetener, none of these, or not sure.

The post-questionnaire was completed following the video. Similar to the pre-questionnaire, participants were asked the same set of questions regarding their knowledge about LNCSs and their confidence regarding their safety and approval process, and to indicate their agreement with several questions/statements relating to the use of LNCSs. Four additional questions were asked regarding the information presented in the video.

#### 2.3.2. Intervention: Educational Webinar

A 1 h video was developed for this study and delivered and recorded by a member of the FDA on the approval process of LNCS. The FDA representative developed the webinar with no specific oversight from the researchers involved in this study. The instructions provided to the FDA were to create a webinar describing the review and approval process of low- and no-calorie sweeteners to be given to healthcare professionals. This presentation was prepared by a member of the FDA and provided a live recording of the talk. A description of the webinar was provided as follows: Health professionals, including dietitians, play a critical role in helping consumers navigate conflicting messages on nutrition and food safety so they can make informed dietary decisions. Recent reports by the World Health Organization (WHO) and conflicting social media messages have added to the consumer confusion about the safety of ingredients added to foods, such as non-sugar sweeteners. This webinar will provide health professionals with a comprehensive overview of the US Food and Drug Administration regulatory review process for food additives, including the food additive petition process and the Generally Recognized as Safe (GRAS) process. Attendees will understand the information reviewed, including intended use, exposure, and safety data, and the standards established by the Food, Drug, and Cosmetic Act (FD&C).

The webinar’s title was as follows: FDA Regulatory Review Process for Food Additives—Including Low- and No-Calorie Sweeteners. Participants could only access the post-screener after completing the entire webinar. This webinar was hosted on the IAFNS website, and a link to the recorded video was provided after completing the pre-questionnaire. The specific performance indicators include the following: (1) interprets and integrates evidence-based research and the literature in decision-making; (2) identifies misinformation and inaccurate information to inform decision-making; (3) interprets and applies evidence-based literature and standards for determining the nutritional needs of targeted audiences. This webinar could be completed at the participants’ leisure, and participants could pause the recording. Participants had the option to receive continuing educational credits after completing the post-questionnaire, which was approved through the Commission on Dietetic Registration for one continuing professional education unit.

### 2.4. Statistical Analysis

Descriptive statistics were performed, and frequencies, means, and standard deviations (±S.D.) were reported. To determine if the webinar influenced perceptions of LNCSs among participants, responses to the questionnaire were compared (pre- vs. post-). The post-questionnaire was completed by 58 participants (a 31% completion rate). To ensure that there were no differences in the pre-questionnaire perceptions of this subset of data, a pairwise two-way *t*-test was conducted to ensure there were no statistical differences in the pre-questionnaire ratings among the total study participants (n = 187) to the participants completing both the pre- and post-questionnaire (n = 58). A pairwise two-way *t*-test was conducted to determine whether there was a significant difference in participants’ responses to questions asked before (pre-) and after (post-) the webinar. All analyses set the alpha to 0.05 and were carried out using IBM SPSS Statistics (Version 29.0.2.0)

## 3. Results

### 3.1. Participant’s Perceptions of LNCS

Participants were asked where they source information or training for new nutritional science research. Participants could select any number of resources, and for each resource selected, a follow-up question was asked regarding how trustworthy they perceived the information from this resource to be (Table 2). The most common resource to access information and/or training was continuing education courses, seminars, or webinars, followed by peer-reviewed journals. Interestingly, peer-reviewed journals were considered more trustworthy compared to continuing education courses.

Next, participants were asked about their experience with LNCSs in their practice. Most participants indicated that about half (36%) or almost all (27%) of their patients ask questions about reducing sugar or added sugar to their diet. Conversely, fewer participants reported that less than half (25.6%) or nearly none (5.3%) of their patients asked questions about sugar and diet, with eight participants (4.3%) reporting that they were not sure. Regarding LNCSs, there was a similar trend, with participants reporting about half (36.4%) and almost all (17%) patients asking questions about LNCSs. Meanwhile, 32.6% and 10.2% reported that less than half, or nearly none of their patients, respectively, asked questions about LNCSs. Patients, too, may have perceptions about the benefits and risks of LNCSs. Therefore, participants were asked whether their patients asked questions about the benefits and risks of LNCSs. More than half (56.1%) indicated that their patients ask questions about both the risks and benefits of LNCSs, with fewer asking questions just about the risks (28.9%) or just about the benefits (4.8%). In comparison, 10.2% reported that their patients do not ask about the risks or benefits of LNCSs.

Participants answered several questions regarding their perceptions and beliefs concerning LNCSs. In the present study, when asked whether they recommend LNCSs to their patients, the majority (43.8%) reported that it depends. In contrast, 41.2% said they recommend LNCSs, and 15.0% do not recommend them to their patients. Participants who indicated “it depends” had the option of describing their views. While answers were diverse, trends were observed. Diabetes was mentioned by 28 participants, indicating that LNCSs were recommended for patients with diabetes. Generally, participants considered other health issues and nutritional goals (e.g., weight loss or other diseases), with some indicating that it was patient-guided.

This study included specific questions about 11 LNCSs. The sweeteners included acesulfame potassium (Ace-K), advantame, allulose, aspartame, neotame, monk fruit, saccharin, stevia, sucralose, sugar alcohols (sugar alcohols or polyols (e.g., xylitol, erythritol)), and thaumatin. Participants were asked to select the sweeteners that they (i) were familiar with, (ii) believed to be safe, and (iii) believed to be unsuitable or lacking safety data. Participants also had the option of reporting all of these, none of these, not sure, or any FDA-approved sweetener (Table 3).

Only two participants reported not being familiar with any of the LNCSs, with five participants reporting being familiar with all the LNCSs. The LNCS familiar to almost all participants was aspartame (92.5%), followed by stevia (90.4%). The least familiar sweetener was thaumatin, with one participant indicating they were familiar with this specific sweetener. Roughly a third (31.5%) of participants reported that they believe all LNCSs that have FDA approval are safe. Conversely, 11.8% indicated that they believe none of these LNCSs are safe.

Regarding the safety of individual LNCSs, stevia was selected as the safest by 64.7% of participants, with the fewest selecting advantame 5.3%. Lastly, participants were asked which LNCSs they believed to be unsuitable or lacking safety data, with the majority (43.8%) indicating that they were not sure and 10.2% responding that all were safe. Of the five participants who responded that they were familiar with all sweeteners, three indicated that all were safe, with one indicating that only stevia was safe, and one responding that none were believed to be safe. A small percentage (8.5%) indicated that either all FDA-approved LNCSs or all LNCSs were not safe or needed more safety data. Based on those who reported being familiar with the specific LNCSs, neotame (88.8%) followed by aspartame (26.7%), these were considered unsuitable or lacked safety data.

Participants answered questions regarding their knowledge, confidence, and perceptions of the LNCS, focusing on the safety and approval process. Responses are reported in Table 3. Most participants (42.8%) indicated they were moderately knowledgeable when asked if they were prepared and knowledgeable about advising patients about LNCSs. Fewer were knowledgeable regarding the safety and approval process for LNCSs, with 31.5% of participants responding that they were somewhat knowledgeable. A similar trend was reported for how much confidence participants had regarding the safety and approval process for LNCSs, with somewhat confident (28.3%) selected most often by participants. Participants then responded as to how much they agreed or disagreed with statements regarding LNCSs. Just over half (53.4%) of participants were confident in their understanding of the safety of LNCSs. A similar result was reported for participants’ trust in the process of determining the safety of LNCSs, with 50.2% indicating they agreed or strongly agreed. The majority (59.9%) indicated they either agreed or strongly agreed that LNCSs are safe to use. Conversely, 44.4% of participants either agreed or strongly agreed that they were concerned that LNCSs may have an unintended or negative impact on health.

### 3.2. Influence of Educational Material on Perceptions of LNCS

After completing the pre-questionnaire, all participants were invited to view a 1 h webinar on the approval process of LNCSs. Upon viewing the webinar, participants completed a post-questionnaire. A total of 58 participants completed the post-questionnaire. The subsequent analysis focuses on this subset of participants to examine how viewing the webinar modified perceptions of LNCSs.

The participants completing the entire study were similar representations of the entire study population. Participants had a mean age of 44 years old (±12.7). The majority reported being an RD (93.1%) and worked with a variety of patient groups: all ages, 34.5%; adult, 31.0%; adult and geriatric, 17.2%; with 10% or fewer seeing pediatric or geriatric groups, while four indicated that they were not seeing patients. Similar to the entire study population, the majority (32.7%) reported practicing for more than 20 years, followed by 5–10 years (24.1%), 11–20 years (22.4%), and 1–5 years (19.0%), with one participant reported to be practicing for less than 1 year.

Most participants (62.1%) reported that their perceptions of LNCSs changed (slightly, somewhat, or greatly) following the webinar. A third (34.5%) reported that they had not changed their opinion of LNCS, and two participants reported that they were not sure.

It was hypothesized that viewing a webinar on the approval process of LNCSs would change perceptions of LNCSs. As a check, the pre-responses of the subset data (pre-; completed pre- and post-questionnaire) were not statistically different from the responses reported by all participants (“All Pre”) (see Table 4). A pairwise two-way t-test was conducted to determine whether there was a significant difference in participants’ responses to questions asked before (pre-) and after (post-) the webinar. The statistical results are reported in Table 4. The results revealed that exposure to the webinar resulted in a significant increase in participants’ knowledge related to advising patients about the safety process of LNCSs and increased confidence regarding the safety and approval process for LNCSs. Participants reported a significant increase in confidence in their understanding of the safety of LNCSs. The results revealed a substantial reduction in the concerns about the unintended or negative impact on health. However, there was no significant change in participants’ responses regarding trust in the approval process or whether they believed LNCSs were safe to use (*p* > 0.05).

In the post-questionnaire, participants answered additional questions regarding their perceptions of the webinar. Fifty-two participants (89.6%) thought the webinar provided new information (agree or strongly agree). In terms of usefulness, 77.6% thought the webinar offered useful information. The majority thought the webinar was either moderately or very helpful (70.7%), with fewer reporting it was somewhat (17.2%) or slightly (12.1%) helpful.

## 4. Discussion

The present study provides essential insights on dietitians’ and nutritionists’ perceptions and knowledge concerning the safety of LNCSs. Perceptions of safety were different across LNCSs, suggesting that some LNCSs are perceived to be safe, whereas others are not suitable or lack safety data. This is interesting, as all LNCSs in the present study currently have approval for use in foods and beverages in the U.S. Stevia was reported by 65% of participants as being safe, followed by sucralose (48%). Conversely, more than a quarter of participants reported that three FDA-approved sweeteners (neotame, aspartame, and saccharin) lacked safety data or were unsuitable. Too few particpants responded to being familiar with thaumatin, suggesting this was the least familiar sweetener, and more work is needed to understand the reason for this lack of familairity. This is the first study to examine dietitians’ and nutritionists’ perceptions of specific LNCSs. Potential drivers of differences in perceptions may be unfamiliar with the safety and approval process of LNCSs. Less than half (43%) reported they were moderately knowledgeable, and 32% reported that they were somewhat knowledgeable regarding advising patients about LNCSs. Regarding the safety and approval process, 53% and 50% were confident or had trust in their understanding of the safety and approval process, respectively. These data are supported by previous studies assessing HCPs’ perceptions of LNCSs. Rossi and colleagues (2019) reported that students receiving training to become HCPs in Brazil reported limited scientific evidence on the health and safety of LNCSs [29]. In a multi-country study, dietitians were uncertain about the negative health effects of LNCSs and felt that there was a lack of guidance on the dietary use of LNCSs [28]. These data highlight a gap in knowledge and understanding among HCPs and support the call for increased nutritional education to improve patient outcomes [35].

Together, prior work suggests a low awareness amongst the dietetic and nutrition provider community on the dietary use of LNCSs. This aligns with prior work, which was previously suggested to be driven by mistrust in government institutions [38,39]. Alternatively, the International Food Information Council concluded from the Food and Health Survey [31] that a lack of trust or understanding of the regulatory processes could be associated with negative perceptions of LNCSs. Farhat and colleagues described a similar finding, noting a lack of knowledge of LNCSs among HCPs, who also described not having access to objective data on the risk–benefit profiles of LNCSs nor being familiar with how they are regulated [30]. The data in the present study align with prior reports that dietitians and nutritionists are unfamiliar with the safety and approval process of LNCSs.

Comparing pre- and post-questionnaire responses among the 58 participants provided evidence that the educational video significantly influenced participants’ opinions on LNCSs. The results demonstrate that understanding of the approval and safety process increased, which may have influenced the respondents’ views regarding using LNCSs. However, when participants were asked if they were concerned regarding the unintended or negative impact of LNCSs on health, responses were significantly improved following the intervention, shifting views away from perceived concerns toward neutral. This work supports prior studies suggesting that dietitians and nutritionists have uncertainty regarding the safety and approval process of LNCSs [30]. However, this cohort has no relationship with the trust in the approval process, as previously suggested [31]. In other words, a lack of trust in the governing body’s approval is not evident but rather a lack of understanding of the approval process. This study provides promising results showing the influence of education on the safety and approval process of LNCSs, helping providers feel more prepared to advise their patients regarding the use of LNCSs. Future studies are needed to understand if this intervention translates to guidance changes.

Interestingly, there was no change in the perception of whether LNCSs are safe to use. This may be due to differences in the perceived safety of individual LNCSs. The post-questionnaire focused generally on all LNCSs. One aspect not assessed in the present study was the reason for LNCSs being perceived as unsuitable or lacking safety data. Factors discussed in the previous literature suggest that whether a LNCS is artificial or natural may influence perceptions of its safety. It is not unique to HCPs that there is inconsistency in the recommendation or use of these two sweetener categories [22,40]. There are inconsistent reports in the literature and across healthcare institutions regarding the safety and recommended use of artificial sweeteners [41,42,43]. The present study included three naturally occurring LNCSs, namely stevia, monk fruit, and allulose. Interestingly, stevia and monk fruit were in the top three LNCSs perceived as safe, whereas allulose had the second-fewest responses considering it safe. However, it is important to note that allulose was among the least familiar LNCSs. While this study did not specifically investigate whether perceived safety was associated with whether the LNCSs were natural or artificial, this may be important to consider in future studies regarding perceptions and dietary recommendations of LNCSs.

It has been advised that education on nutrition for clinicians will positively influence patient health [35], and continuing educational programs could be an effective way to provide educational information regarding nutritional guidance [44]. The present study suggests that for LNCSs, continuing educational programming can positively influence dietitians’ and nutritionists’ confidence regarding advising patients on using LNCSs. However, it is essential to note that of the 187 participants who completed the pre-questionnaire, roughly one-third completed the post-questionnaire. While most participants indicated that the information presented was new, it is uncertain whether the perceived novelty of the information given was associated with completion. In other words, participants who found the webinar to be informative and valuable were more likely to complete the post-questionnaire. Due to the low completion rate, it is also unclear whether continuing educational programming presented through a webinar is an effective strategy. Interestingly, participants in this study indicated that the most trustworthy resource for accessing nutritional information (i.e., very reliable) was through peer-reviewed journals, followed by continuing educational courses/webinars and professional association meetings. Thus, future educational programming may consider continuing educational programming, peer-reviewed literature, and professional association groups as potential opportunities to present new nutritional science research, as HCPs find these to be the most trustworthy.

This is the first study to assess perceptions of LNCSs and quantify the effectiveness of education information on perceptions among dietitians and nutritionists in the U.S. While this study has highlighted the differences in perceived safety across LNCSs and that information on the safety and approval process of LNCSs can increase knowledge and confidence in advising patients on the use of LNCSs, it is important to note the limitations of the study. This cohort is a relatively small sample size consisting of mainly registered dietitians and it is not appropriate to translate the findings to all HCPs. One reason for the lack of recruitment of diverse HCPs is that this information on LNCS attracted dietitians and nutritionists as it was more relevant to their practice. This suggests that dietitians and nutritionists may receive more concerns or questions from patients regarding the use and safety of LNCSs. Furthermore, there was a high loss of follow-up, with one-third of the participants completing the post-questionnaire. As mentioned above, this raises questions on whether all participants found the educational content to be novel and whether webinars are an effective tool for presenting nutritional information to participants. The post-questionnaire assessed perceived knowledge and confidence and was not intended to evaluate learning. Furthermore, this study cannot assess whether the educational information delivered will influence HCPs recommendations and changes in patient behaviors.

## 5. Conclusions

The present study suggests that dietitians and nutritionists have differing perceptions of LNCSs, with some sweeteners perceived to be unsuitable or lacking safety data, despite the fact that all of the LNCSs had received approval for use in foods and beverages. Out of the 11 LNCSs included in the present study, only one sweetener was perceived to be safe by more than 50% of participants. More research is needed to determine what factors influence perceptions of LNCSs, as there was no trend to suggest these perceptions were driven by the natural or artificial origins of LNCSs. The present study determined that education on the approval and regulatory process of LNCSs can effectively influence perceptions of LNCSs, resulting in increased perceived knowledge and confidence in the approval process and providing guidance to patients. Webinars and educational tools may be an effective strategy for increasing knowledge related to LNCS, which may translate to improved consistency in patient guidance. There is a need for further studies to document the perceptions of LNCSs and whether these educational strategies increase consistency in patient recommendations. This information is anticipated to assist HCPs in making objective recommendations during their clinical practice, improving confidence and patient guidance to support health outcomes.

## Figures and Tables

**Table 1:** Perceptions, concerns, and recommendations of HCPs on dietary use of LNCSs for health and disease management (e.g., diabetes, obesity, and weight loss).

Reference	Title	Target	Conclusions on Perceptions, Concerns, and Recommendations	Country
Harricharan, Wills [28]	Dietitian perceptions of low-calorie sweeteners	Dietitians, n = 75	Dietitians’ perceptions were uncertain, ambivalent, and divergent, with fears about adverse health effects. Clear guidance required on use of LNCSs in dietetic practice.	France, Germany, Hungary, Portugal, UK
Rossi, Bruno [29]	Guidance on Healthy Eating Habits from the Medical Student’s Perspective	Medical students, n = 28	There is limited scientific evidence on health and safety, which is contradictory and inconclusive. HCPs should be up to date with the scientific evidence, advantages, disadvantages, and recommended ADIs for LNCSs.	Brazil
Farhat, Dewison [29]	Knowledge and Perceptions of Non-Nutritive Sweeteners Within the U.K. Adult Population	Mixed adult population, n = 1589, HRPs = 209	HRPs had similar levels of concern and lack of information as the public. Associations of LNCSs with cancer, gut microbiota disruption, hormonal disturbances, and long-term risks. Evidence is scant and there is high-risk perception despite the role of government health agencies and regulatory bodies.	UK

HRPs—health-related professions; LNCSs—low- and no-calorie sweeteners.

**Table 2:** Resources participants use to access information or training on new nutritional science research. Number (%) reported for participants selecting resources and how trustworthy the information is from that resource.

		How Trustworthy Is the Information?				
Resource	Used	Not at All	Somewhat Not	Neither	Somewhat	Very
Continuing education courses/webinars	179 (95.7)	-	4 (2.1)	3 (1.6)	77 (41.2)	95 (50.8)
Peer-reviewed journals	132 (70.6)	-	4 (2.1)	2 (1.1)	25 (13.4)	101 (54.0)
Professional association meetings	124 (68.2)	2 (1.1)	2 (1.1)	2 (1.1)	28 (15.0)	82 (43.8)
Formal education	49 (26.2)	-	-	2 (1.1)	14 (7.5)	33 (17.6)
Key opinion leaders in your network	29 (15.5)	-	4 (2.1)	1 (0.5)	17 (9.1)	8 (4.3)
Social and traditional media reports	39 (20.9)	5 (2.7)	17 (9.1)	1 (0.5)	14 (7.5)	2 (1.1)
Trade association communications	26 (13.9)	-	4 (2.1)	1 (0.5)	15 (8.0)	6 (3.2)

**Table 3:** The number (%) of participants selecting each LNCS with regards to being familiar, perceived to be safe, and perceived to be unsuitable or lacking safety data for all participants (n = 187).

		Perceive to Be *	
LNCS	Familiar with	Safe	Unsuitable/Lack Safety Data
Ace-K	97 (51.9)	41 (21.9)	33 (17.6)
Advantame	12 (6.4)	10 (5.3)	19 (10.2)
Allulose	57 (30.5)	33 (17.6)	20 (10.7)
Aspartame	176 (94.1)	68 (36.4)	50 (26.7)
Monk fruit	133 (71.1)	83 (44.4)	20 (10.7)
Neotame	18 (9.6)	8 (44.4)	7 (38.8)
Saccharin	152 (81.3)	54 (28.9)	41 (21.9)
Stevia	172 (92.0)	121 (64.7)	18 (9.6)
Sucralose	164 (87.7)	89 (47.6)	27 (14.4)
Sugar alcohols	159 (85.0)	70 (37.4)	28 (15.0)
Thaumatin	1 (0.5)	1 (0.5)	0 (0)

* Perceived to be [safe] or [unsuitable/lacking safety data] were only asked if participants reported being familiar with the sweetener. Thus, percentages are based on the number of participants reporting familiarity with that specific LNCS.

**Table 4:** HCPs’ perceptions and knowledge of the safety and approval of LNCSs.

	All Pre	Pre	Post	
Question	n = 187	n = 58		*p*-Value
How prepared and knowledgeable do you feel advising your patients/clients about LNCSs?	3.7 (±0.92)	3.7 (±0.99)	4.0 (±0.93)	0.004
How knowledgeable are you regarding the safety and approval process for LNCSs?	2.9 (±1.13)	2.9 (±1.12)	3.9 (±0.94)	<0.001
How much confidence do you have regarding the safety and approval process for LNSCs?	3.0 (±1.24)	3.2 (±1.19)	3.8 (±1.21)	<0.001
I trust the approval process for determining the safety of LNCSs	3.5 (±0.87)	3.5 (±0.88)	3.7 (±0.99)	0.375
I feel confident in my understanding of the safety of LNCSs	3.5 (±0.80)	3.5 (±0.88)	3.8 (±0.87)	0.014
I believe that LNCSs are safe to use	3.6 (±0.90)	3.6 (±0.88)	3.7 (±0.94)	0.461
I am concerned that LNCSs may have unintended or negative impact on health *	2.8 (±0.98)	2.7 (±0.94)	3.1 (±0.98)	0.007

Mean values (±SD) are reported. Responses were collected on a 5-point Likert scale and recoded 1 to 5 (refer to Section 2.4 for details on the recoding). Briefly, higher values (5) indicate agreement. * The question was reverse-coded. “All Pre” refers to all participants (n = 187) who completed the pre-questionnaire. A paired *t*-test was conducted to compare the differences in the pre- and post-questionnaire results for the subset of participants who completed both questionnaires (n = 58).

## Data Availability

The data presented in the study will be made available on request from the corresponding author.
